# Osborn waves in a hypothermic patient

**DOI:** 10.3402/jchimp.v1i4.10742

**Published:** 2012-01-26

**Authors:** Sami W. Serafi, Crystal Vliek, Mahnaz Taremi

**Affiliations:** 1 Union Memorial Hospital, American University of Antigua, Baltimore, MD, USA; 2 Union Memorial Hospital, Department of Cardiology, Baltimore, MD, USA; 3 Union Memorial Hospital, Department of Medicine, Baltimore, MD, USA

**Fig. 1:**
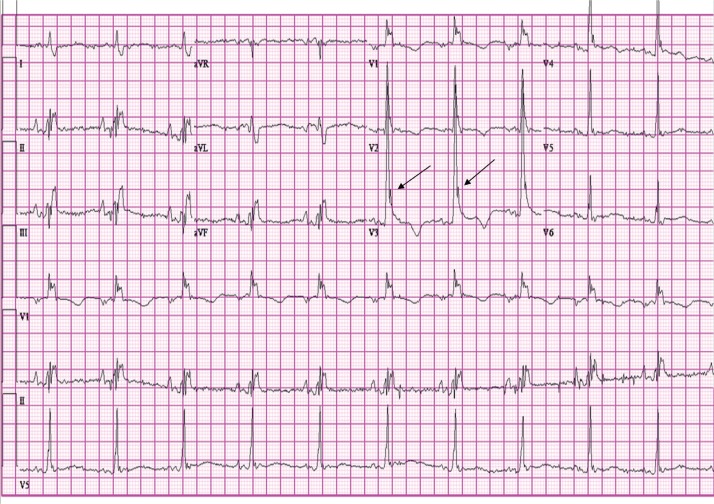
Osborn waves on admission ECG can be seen clearly in the precordial leads. Arrows point to Osborn waves.

**Fig. 2:**
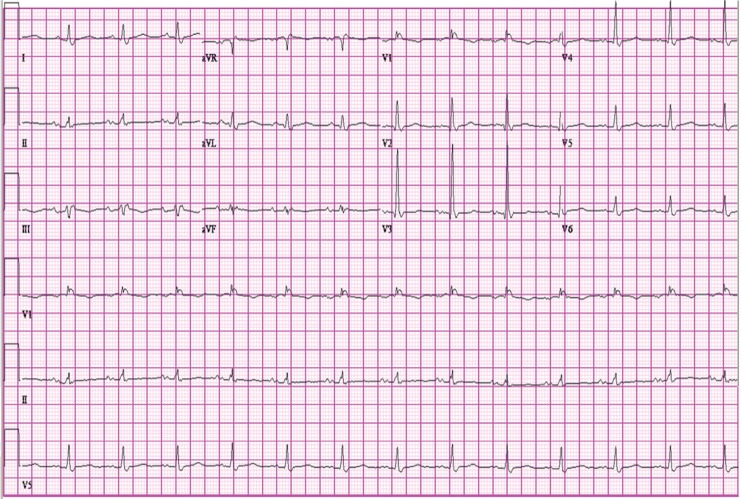
Osborn waves resolved 48 hours after admission.

A 56-year-old man presented with hypothermia (rectal temperature of 30.1°C) and hypotension after being found by emergency medical services (EMS) on his basement floor. A 12-lead electrocardiogram (ECG) revealed normal sinus rhythm with a rate of 62 bpm, right bundle branch block, prolonged QT interval (QTc of 564 ms), wide QRS (110 ms), and a prominent J wave in the precordial leads (Fig. 1). After the patient was warmed to a normal core body temperature, hydrated, and made normotensive, repeat ECG showed a normal sinus rhythm of 79 bpm, right bundle branch block, prolonged QT (QTc of 488), and resolution of the J waves (Fig. 2).

The J wave is also known as an Osborn wave, camel-hump sign, late delta wave, hathook junction, and hypothermic wave (1). The prominent J deflection attributed to hypothermia was first reported in 1938 by Tomaszewski. Over time, the wave has increasingly been referred to as an Osborn wave, in most part due to Osborn's 1953 article in the *American Journal of Physiology* on experimental hypothermia (2).

An Osborn wave is characterized as an extra deflection of the terminal junction of the QRS complex and the start of the ST segment (3). Typically, the deflection at the J point is in the same direction as that of the QRS complex (4). It is more commonly observed in leads II, III, AVF, V5, and V6. The J wave disappears with normothermia (5). This deflection has been attributed to delayed depolarization, to a current of injury, or to early repolarization. In leads that face the left ventricle, the deflection is positive and its size is inversely related to body temperature (6). The earliest morphologic abnormality in patients with mild hypothermia is a tremor artifact due to the shiver response. This is non-specific and becomes uncommon at core body temperatures less than 32°C as the body's ability to generate a shiver response diminishes. As core body temperature approaches moderate hypothermia, we find the appearance of the J waves. J waves can be considered highly suggestive of hypothermia but are not considered to be pathognomonic (3). Conditions other than hypothermia have been reported to cause an abnormal J wave deflection such as hypercalcemia, brain injury, subarachnoid hemorrhage, damage to sympathetic nerves in the neck, and cardiopulmonary arrest from oversedation (2). A deflection similar to the J wave is also present in patients with Brugada syndrome (7). J waves have no relationship to pH, sodium, potassium, or chloride concentrations (3).
